# The Necessity for the Filtration of a Polluted Public Water Supply1Presidential address read at the forty-second annual meeting of the Medical Association of Central New York, held at Auburn, October 19, 1909.

**Published:** 1910-03

**Authors:** M. P. Conway

**Affiliations:** Auburn, N. Y.


### The Necessity for the Filtration of a Polluted Public Water Supply[1]

By M. P. CONWAY, M. D., Auburn, N. Y.

THE long line of distinguished men who have occupied, since the installation of Dr. Edward Mott Moore, of Rochester, in 1868, as the first president of this society, the chair that I hold today, presented in their annual addresses either a subject "which dealt with the history of medicine and traced the source of our power," or they gave you a resumé of the advances made in medicine and surgery during the intervening time of your meetings.

I shall depart somewhat today from this practice that has usually obtained, and I shall offer for your consideration a topic that is engrossing the attention of sanitarians and physicians in every city, village and hamlet in this broad land—namely, The Necessity for the Filtration of a Polluted Public Water Supply.

For the purpose of pointing out more clearly the advanced and modern ideas that direct and control this important public health problem, and because I believe the conditions are typical of those that exist in many other places, I shall make especial reference to the water supply of the City of Auburn, and in this connection I shall outline in a brief way, the results of an in-

1. Presidential address read at the forty-second annual meeting of the Medical Association of Central New York, held at Auburn, October 19, 1909.

vestigation made of a small epidemic of typhoid fever that occurred here last year and which was attributed to the water.

This subject of my address may be construed as an indication that the field of work of the physician is year by year broadening and that only by ceaseless study and observation will we be able to keep abreast of the knowledge of our time. As further proof of this, I wish to cite the fact that, within very recent time there has been established at Harvard, a Department of Preventive Medicine and Hygiene. The September number of the graduates magazine of that university announces the birth of this new department as follows:

As its name indicates, it has for its field of work the laws of health in relation to the prevention of the occurrence and the limitation of the spread of disease; it will consider the laws of the town, the state and the country in their bearing on the health of the community, and the natural history of disease in relation to the individual and the community; it will train men for the investigation of these problems, and men to fill various offices in boards of public health and other public health work; it will meet the growing need for men to direct the people in ways of rational healthy life.

It is obvious that no discussion of the subject which I have selected can be carried very far without injecting the time-worn but always interesting theme of typhoid fever. The elusive bacillus of Eberth has generally been regarded, since its discovery in 1880, as the cause of this now, so-called American disease. If there are those who question that this bacillus is the specific cause of typhoid fever, let us reply that the lower animals such as horses, dogs and cats are not susceptible to the disease and that the Eberth bacillus "can be isolated from persons sick or who have been sick with typhoid fever, and from those persons only." We know positively that it is present in enormous numbers in the blood of our typhoid patients and that at this time it invades almost every organ of the human body. Further, it is a fact, not now disputed, that some persons who are in apparent good health discharge typhoid bacilli in live and virulent form in their urine and feces for periods of months and years after an attack. It must therefore be plain that "bacillus carriers" may be particularly active agents in spreading the infection throughout a community.

Not long ago I saw that the health authorities of New York City had committed for an indefinite period to a detention hospital a woman, known as Typhoid Mary, a cook, who in good health, and changing from place to place left, as has been said, a trail of twenty-eight cases in the houses where she had served.

The commitment made in this case, while primarily for the purpose of preventing her from infecting others, was also made with the idea that by some course of treatment she might eventually cease to be a "carrier." Fortunately the percentage of this class of cases is small, the large majority becoming free of the bacilli a short time after recovery.

There is no better index of the general sanitary condition of a public water supply than the typhoid fever death-rate of a community supplied by it. This is especially true of large cities where the death-rate is less likely to be influenced by local epidemics due to causes other than water. Fortunately, typhoid fever is becoming less frequent as a cause of death. This is due in part to the methods used by physicians in treating this disease, but it is believed to be chiefly due to the better water supplies that the authorities are giving today to the public.

One can travel through England, Belgium, Holland and Germany and drink water at every city visited without any anxiety as to its effect upon his health. But this has not always been so. In 1892, in that great important commercial center of Germany, Hamburg, having a polluted water supply, like hundreds of our American cities have today, there were sacrificed 8,000 lives in one month for this carelessness. Since that time Germany has spent millions upon millions in her efforts to secure pure drinking water for her cities, with this result, that her low typhoid rate today astonishes us. Another reason for this healthy condition in Germany is that the filtration of all surface waters is required by law and rigid restrictions are in force as to the efficiency necessary to be obtained by the filters, compulsory reports being made of the bacterial character of the water before and after filtration. In Japan the water supplies of most of the large cities have been subjected to filtration for a number of years. It might not seem inappropriate to say just here that, according to the reports of the representatives of the American army, typhoid was less prevalent in the Japanese army during the war with Russia than in many American cities. This result was obtained because of the care they exercised to secure pure drinking water, and to properly dispose of fecal matter, and because of their campaign against flies.

If these things be true, and there is no evidence to contradict them, then are we not less advanced in this line of work than some of those countries, an invitation to compare ourselves with which would cause a blush of shame to come to our cheeks. One can easily see why continental Europe calls typhoid fever an American disease. And we, ourselves, should therefore be not surprised, as we visit the intelligent centers of our American civilisation, which have concededly polluted water supplies, to see in the daily newspaper, the glaring headlines of warning: "Boil the water; *Boil* the water."

I shall not dwell upon the waterborne epidemics of the last few years, with which you all are undoubtedly as familiar as the writer. I shall only mention in passing the names of Ithaca, Watertown and Albany, in our own great state; Pittsburg, Allegheny, Scranton, Plymouth and Reading in Pennsylvania; Lowell, Lawrence and Springfield in Massachusetts; Stamford and New Haven in Connecticut, and Waterville and Augusta, in Maine; populous cities where typhoid has dealt hard blows through the drinking of polluted water. Cleveland, in the west, made a record a short time ago with her 4,578 cases and 611 deaths from typhoid fever, due to the fact that her citizens drank the water from Lake Erie that had been polluted with sewage from their own city. The drinking of water taken from the Detroit River, at a point not far from where the sewage of that city enters, was the undoubted cause of an outbreak of typhoid fever among the passengers on one of the large passenger steamers running between Buffalo and Chicago. Auburn had its representatives on this unfortunate list. And who is there among us, as memory retraces her steps, who is not able to recall the sacrificed life of one of our number who died from this preventive affection because of the drinking of the polluted Hudson River water while attending a session of our State Society at Albany.

Typhoid fever is by no means the only disease transmitted by contaminated water. Dysentery and various other diarrheal diseases are frequent as a result of drinking water of this character. Recently the question has been raised again whether or no tuberculosis can be conveyed by drinking water, infected largely by the slope washings of fields covered for fertilisation purposes by the manure of tuberculous cattle. I have not been able to find any satisfactory evidence to uphold this theory, but surely the intestinal discharges of diseased cattle will not improve the quality of a water.

Water is not the only medium, as we know, of typhoid epidemics. Schüder collected from the literature 650 typhoid epidemics, the supposed cause of which had been reported; 462 were reported by him as spread by water and 110 by milk. Trask reports 179 typhoid epidemics as spread by milk, 107 of which occurred in the United States.

### WATER SUPPLY OF AUBURN.

Auburn, the city which today entertains you, including its large class of undesirable citizens who occupy state rooms each night, has approximately 40,000 inhabitants. The source of the water supply of this city is Owasco Lake, distant two miles, a beautiful sheet of water about eleven miles long, about one mile in width, with a depth of 177 feet at its deepest point. The watershed covers an area of 204 square miles, is about 30 miles north and south, and 10 miles east and west; upon this watershed there dwells a population of about 10,000 persons. The sloping lands which form in great part this watershed are the fertile farms of the thrifty people whose horses and cattle are found in considerable number on either side of the lake, the intestinal discharges from which, especially after heavy rains or thaws, are recognised in our water by the presence of bacillus coli. By reference to the bacteriological reports of the local water board I find that these organisms are always found after these thaws and rains, and not only that, but I also find that at such times the count of all form of bacteria takes a rapid rise. And what is of some significance, these reports also show that some of these high bacterial counts have occurred when the lake is frozen.

Concerning the significance of bacillus coli in drinking water, I find that there is much difference of opinion. Kruse asserted that "this organism is so ubiquitous that it can not be regarded as characteristic of sewage," and in this position he has received the support of a number of other investigators. The committee of the laboratory section of the American Public Health Association however reported not long ago that "the colon test of water is a safe index of pollution, and that their number rather than their presence should be used as a criterion of recent sewage pollution." The general conclusion in this matter is, however, that the presence of this organism is proof of pollution of the water from animal sources, but not necessarily from human sources.

Included in the number of 10,000 people, of which I made mention a moment ago, are the inhabitants of several villages upon streams that flow into the lake, the largest of which are Groton, Locke and Moravia. While there is no general sewage system in these places, and therefore no disposal plants, one can observe, on every side, sewage pouring through drains into the streams that eventually form the inlet of our lake. Moravia, the largest of these villages, a place of 2,000 inhabitants, has several sewers in her main street which discharge directly into the inlet three miles above our lake.

### AUBURN'S TYPHOID EPIDEMIC.

In the fall of 1907 twenty cases of typhoid fever occurred in towns within the drainage area of the Owasco Lake watershed. An effort was made by the local authorities, working in harmony with the health officers of the several towns, to prevent any typhoid infectious material reaching the lake. During the early winter there was no evidence of any infection of the water, nor was there any persistent increase in the number of bacteria, the analysis of November 1st, showing only 8 bacteria per c. c. About the middle of February, thaws and rains caused the lake level to rise with the result that on February 19 and 23, the count of bacteria had risen to 4,000 and 3,300 respectively, with bacillus coli present. Between March 1 and July 20, following (1908), Auburn had 45 cases of typhoid fever develop. Twenty-seven cases or 60 per cent. of the entire number occurred during the month of April. The 45 cases occurred in the practice of 25 physicians. There were 30 cases among males and 15 among females. The census showed that within twenty days preceding attack, 39 of the cases had used city water, unboiled. City water is used almost exclusively in Auburn. The census further showed that 12 deaths occurred, making a mortality rate of 26.6 per cent. In a series of 65 bacteriological examinations made between the above dates (March 1 to July 20) bacillus coli were found in 20 per cent. of the analyses made of the lake water. No bacillus typhi were isolated.

During the time of this epidemic, differences of opinion arose as to the source or origin of the infection and the arguments that followed, pro and con, were loud and long. Even in the medical profession honest differences of opinion were entertained and forcibly expressed.

EPIDEMIC INVESTIGATED.

For the purpose of determining whether any possible relation existed between the epidemic and the water supply, two firms of sanitary engineers were employed and an exhaustive investigation followed. Cross sections of the lake were made, and the depths at various points having been recorded, the capacity of the lake was learned; the amount of water discharged for a stated period was found from the records; the linear velocity of an average particle of lake water was determined and the time required for such particle to pass the length of the lake was ascertained; the rainfall, the prevailing direction of the wind and the atmospheric temperature records were all considered and a careful examination of the location of each case of typhoid of the preceding fall, together with the disposal made of the dejecta, was studied. There was also a study of the conditions and circumstances attending each individual case of typhoid in Auburn; a study of the occurrence of typhoid here during several years preceding 1908, and, finally, a partial inquiry into the supply of milk and dairy products.

WATER SUPPLY AT FAULT.

One of the conclusions reached, in the separate reports made, was that the data did not enable the particular case or cases that gave origin to the epidemic to be determined, but the opinion was expressed that the circumstantial evidence that the outbreak was due to the water was so strong as to make this conclusion practically certain. It was shown that under certain conditions it is possible for the sewage contamination at Moravia, Locke and Groton to reach the intake pipe of the Auburn water works. This latter opinion, given by men than whom none in any country are more competent to speak, was positive in its character. It disposed of that old idea, at least as far as bacteria are concerned, that water purifies itself in running a certain distance. The established fact was pointed out that the effect of sunlight, aeration, and the like, are comparatively unimportant and that the principal agencies that tend to reduce the number of any pathogenic germs that get into a water supply are dilution, sedimentation and the natural death of the bacteria. The idea that water entering a lake merely displaces a certain amount of water, pushing out the water in front of it, is apparently not true and we are told that it never occurs partly on account of thermal stratification and partly because the wind sets up currents in the direction in which it blows.

With these conclusions hereinbefore named, came the opinion that "the only safe and thoroughly satisfactory method to maintain the purity of the water supply is to filter the water;" and it was recommended that a filtration plant be installed. There was no hesitancy under these circumstances on the part of these who, under the law and by virtue of their oath of office, are charged "to procure at all times a pure and wholesome supply of water;" plans and specifications were immediately ordered and they are now nearing completion.

Filtration was first adopted in connection with municipal water supplies in the year 1829 at London, England. In its earlier years its sole function seems to have been the removal of visible suspended matters and the consequent improvement in the appearance of the water. The germ theory of disease stamped a new phase on the question of filtration. It is now known to be able to remove not only disease-producing germs but also all tastes and odors. As ordinarily practised today it consists essentially in passing water downward through beds of sand of medium-sized grains, the sand layers being contained in water-tight basins, and supported on graded layers of gravel. Usually the sand layer is three or four feet thick and the gravel about one foot. In a climate like New York, it is customary to cover these filters to protect them from freezing weather. The modern filtration plant leaves no room for doubt as to its efficiency, notwithstanding the occasional reference made to the prevalence of typhoid in some places where filtered water is used. *Nor will*

*the employment of any number of men, as sanitary inspectors on a watershed, eliminate the danger* to the health and lives of a people who drink a water which is admittedly polluted by the sewage of villages at the source of the supply.

IN CONCLUSION.

We are profiting today by the experience of the old world which led us in this work and which tells us that deaths from water-borne diseases there have been reduced almost to a minimum. One may easily grasp the significance of this when told that in Europe there are now more than twenty-five millions of people supplied with filtered water. Since the time that Lawrence built its first filter in 1893 to care for its polluted Merrimack water down to the present time, when Springfield in the same state is expending over two millions of dollars for its new supply and its filters many other American cities have recognised the wisdom and the necessity of filtering their water supplies. During the last five years, no less than fifty of our cities have undertaken work of this character, and in every instance the efficiency of filtration has been demonstrated.

One by one our American municipalities are falling into line and taking up this important work. Wherever one may travel, north, south, east or west, from Winnipeg in far distant Manitoba to Providence in the east, one will find there the question of the Necessity for the Filtration of a Polluted Public Water Supply has been affirmatively answered.

